# Diuretin

**Published:** 1890-06-14

**Authors:** 


### DIURETIN.

Under this designation Messrs. Knoll and Co., of Ludwigshafen, have, at the suggestion of Dr. van Schroeder, of Strassburg, and Dr. Gram, of Copenhagen, introduced a new form of theobromine, viz., Theobromine-sodio salicylate. Theobromine is closely allied to caffein, these bodies being respectively di- and tri-methylxanthine, but while they both act as diuretics, theobromine does not, like caffein, excite the central nervous centres and induce sleeplessness. Theobromine, however, in the free state is extremely insoluble, requiring 1,600 parts of water for its solution, and is apt to cause vomiting by its direct action on the stomach. The compound salt in question is soluble in half its weight of water, and is free from these objections, and though containing 50 per cent. of theobromine, is much cheaper than the pure alkaloid.

